# Supplementary material for: SWELL1 is a glucose sensor regulating β-cell excitability and systemic glycaemia
Source: Nat Commun. 2018 Jan 25;9:367. doi: 10.1038/s41467-017-02664-0 (PMC5785485; doi:10.1038/s41467-017-02664-0)
Supplement: Supplementary file 1 — Supplementary Information [file 41467_2017_2664_MOESM1_ESM.pdf]

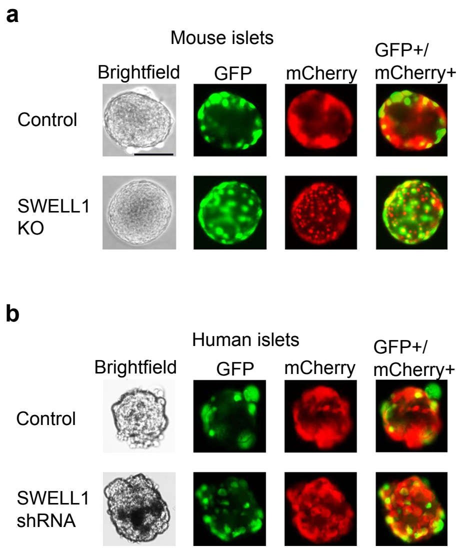

Supplementary Figure 1. Fluorescence images of adenovirally transduced murine and human islets. (**a**) Murine islets freshly isolated from *Swell1*$^{fl/fl}$ mice, cultured (BF: Bright field) and then co-transduced with Ad-RIP2-GFP (GFP) and Ad-CMV-mCherry (top, cytosolic mCherry: Control) or Ad-CMV-Cre-mCherry (bottom: nuclear-localized Cre-mCherry fusion protein; *Swell1* KO). (**b**) Human islets cultured (BF: Bright field) and then co-transduced with Ad-RIP2-GFP (GFP) and Ad-U6-shSCR-mCherry (top, mCherry: Control) or Ad-U6-shSWELL1-mCherry (bottom: mCherry; SWELL1 KD). Scale bar represents 50 μm.

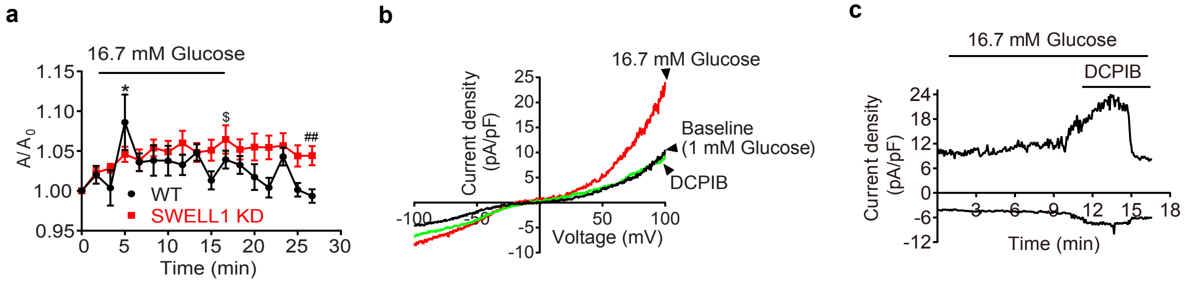

Supplementary Figure 2. Human β-cell $I_{Cl,SWELL}$ is activated by physiological swelling in response to glucose stimulation.
(**a**) Cross-sectional area of primary WT (n = 9 cells) and SWELL1 KD (n = 8 cells) human β-cells in response to glucose-stimulation (16.7 mM glucose). (**b-c**) Human primary β-cell VRAC current-voltage relationship (**b**) and over time (**c**) with DCPIB inhibition (10 μM). In (a), *p < 0.05 vs 0 min in WT, paired t-test; $^{\$}$p < 0.05 vs 0 min in SWELL1 KD, paired-test; $^{\#\#}$p < 0.01 WT vs SWELL1 KD, unpaired t-test.

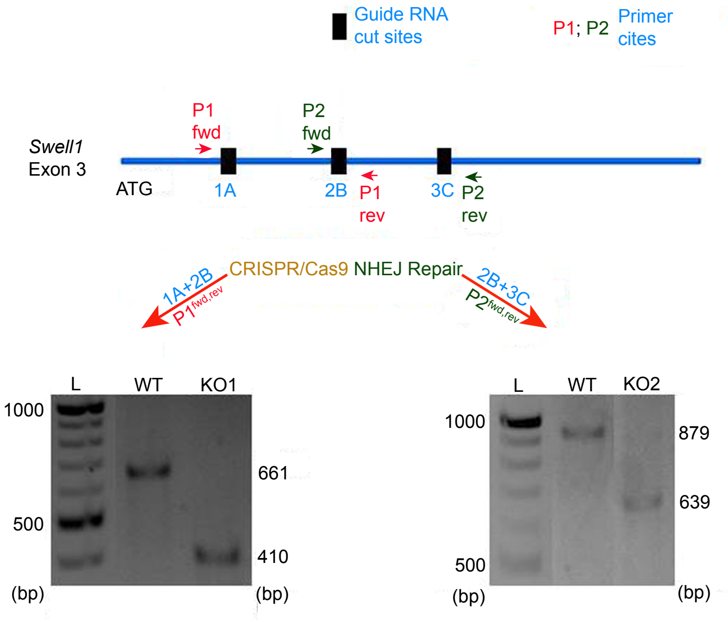

Supplementary Figure 3. CRISPR/cas9-mediated *Swell1* ablation in MIN6 cells Guide RNA sequences targeting exon 3 of the Swell1 gene were used in combinations of either 1A+2B or 2B+3C to generate KO1 and KO2 clones respectively. Upon interacting with cas9 enzyme and corresponding guide pairs the target region undergoes non-homologous end joining (NHEJ) repair. This results in the deletion of DNA base pairs in-between the two target guide sites. Using specific primers for the regions flanking the two target guide sites, the wild-type, WT (non-transfected) cells generate a fragment of size 661 and 879 bps for the 1A/2B and 2B/3C sites respectively, upon PCR amplification. The KO1 and KO2 clones (transfected) generate a deleted DNA fragment of size approximately 410 and 639 bps for the 1A/2B and 2B/3C sites respectively. In the agarose gel image, the DNA fragment sizes are indicated in base-pairs (bp) and ‘L’ indicates ladder.

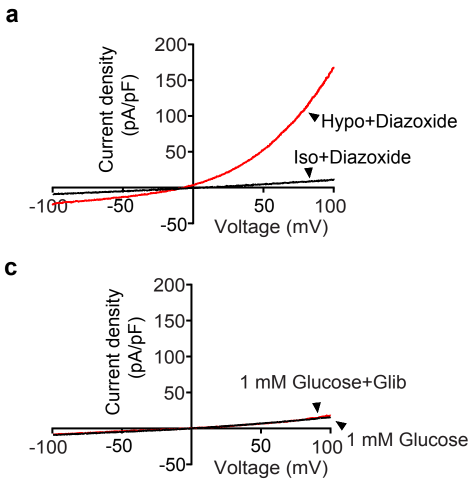

Supplementary Figure 4. Diazoxide and glibenclamide effects on $I_{Cl,SWELL}$ (**a**) Representative current-voltage relationship and (**b**) current-time relationship of $I_{Cl,SWELL}$ in WT murine β-cell at baseline (black trace) and after perfusion with diazoxide (100 μM) upon hypotonic stimulation (210 mOsm, red trace). (**c**) Representative current-voltage relationship and (**b**) current-time relationship of $I_{Cl,SWELL}$ in WT murine β-cell in response to 1 mM glucose (black trace) and 1 mM glucose plus glibenclamide (10 μM) (red trace). Each recording is representative of those from four separate experiments.

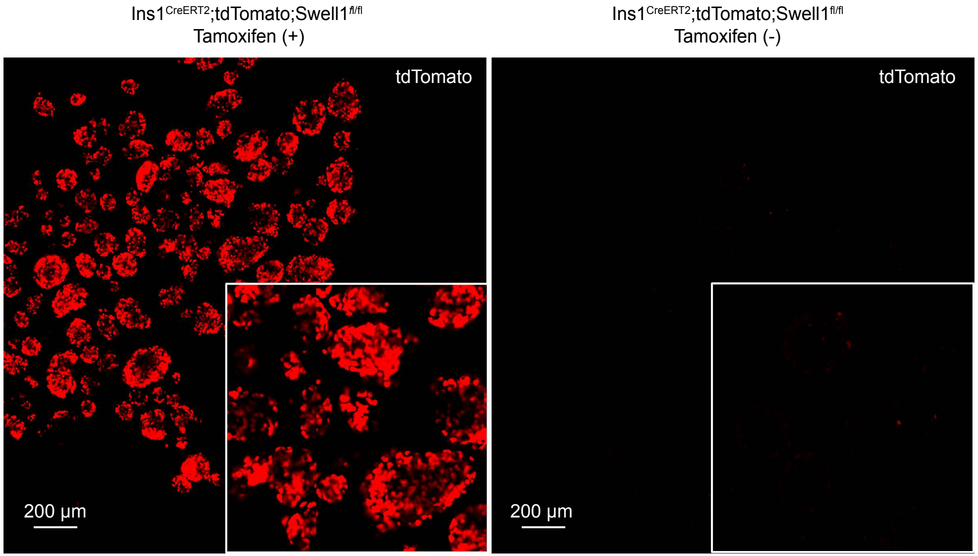

Supplementary Figure 5. Tamoxifen-induced expression of tdTomato in β-cells Tamoxifen-induced expression of tdTomato in β-cells within islets isolated from *Ins1^{CreERT2};Rosa26-tdTomato;Swell1*$^{fl/fl}$ mice. *Ins1^{CreERT2};Rosa26-tdTomato;Swell1*$^{fl/fl}$ mice were injected with 80 mg/kg tamoxifen five times over a 2 week period. Tamoxifen-treatment induced robust β-cell restricted tdTomato expression (red) (left, enlarged in inset) while tdTomato expression was not detected in untreated mice (right). Scale bar represents 200 µm.

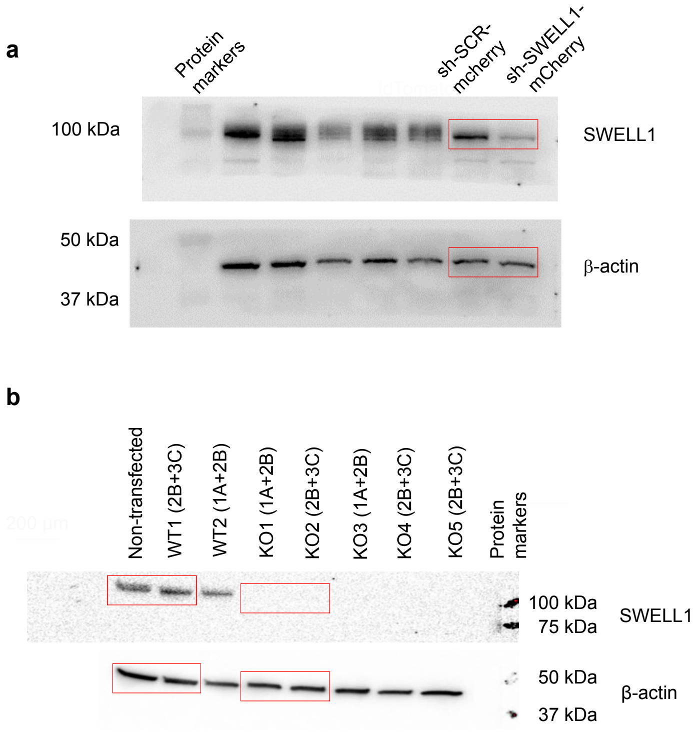

Supplementary Figure 6. Original blots (**a**) Original western blot in MIN6 cells transduced with sh*Swell1* compared to scrambled short-hairpin RNA (shSCR). β-actin was used as loading control. (**b**) Original western blot in WT and CRISPR/Cas9-mediated *Swell1* KO MIN6 cell lines. Red rectangles indicate specific bands shown in the corresponding text figures.

**Vector**	**Target region**
1A	CCTGCAACGACTCCTTCCGGGG
2B	CCACGCACCAGTTCGAAGCTGG
3C	CGATCGGAGACGGGCGTACTGG
